# Coronal restoration of the root filled tooth – a qualitative analysis of the dentists' decision‐making process

**DOI:** 10.1111/iej.13442

**Published:** 2020-12-04

**Authors:** V. S. Dawson, H. Fransson, E. Wolf

**Affiliations:** ^1^ Department of Endodontics Faculty of Odontology Malmö University Malmö Sweden; ^2^ Department of Endodontology Institute of Odontology Sahlgrenska Academy University of Gothenburg Gothenburg Sweden

**Keywords:** crowns, decision‐making, dental restoration, endodontics, permanent, qualitative research

## Abstract

**Aim:**

To describe the decision‐making process of the general dental practitioner (GDP) underlying the choice of coronal restoration of a root filled tooth.

**Methodology:**

GDPs were strategically selected with respect to gender, age, undergraduate dental school, service affiliation and duration of professional experience. Semistructured in‐depth interviews were conducted, focusing on the informant’s personal experience of the process which leads to a decision as to how to restore a root filled tooth. The informants were invited to describe in their own words the latest two cases involving decisions of direct or full crown restorations respectively. The interviews were digitally recorded and transcribed verbatim. Interviews from 14 informants, aged 27–64 (mean age 46 years), were included and analysed according to Qualitative Content Analysis.

**Results:**

A theme (latent content) was identified: Clinical factors were considered important but were overruled by context and patient opinions, if in conflict. Three main categories, including seven subcategories (manifest content), were identified. The categories were clinical factors, contextual factors and patient's views. Clinical factors underlying the GDPs' decision included the current dental status and the estimated longevity of the intended restoration. In certain cases, contextual factors were also of importance, either supporting the GDPs' decision or modifying it. However, the patient's views played a decisive role in the final choice of coronal restoration, leading to either mutual acceptance or a compromise, taking into account the patient's economic status and opinions.

**Conclusions:**

With respect to coronal restoration of a root filled tooth, the GDPs’ decision‐making process was based not only on clinical factors, but also on decisive contextual factors and consideration of the patients’ views.

## Introduction

A determining factor for the successful outcome of root canal treatment (RCT) is a coronal restoration of adequate quality (Ng *et al*. 2008, 2011a). Regardless of whether a direct or an indirect restoration is chosen, the aim is to restore the function and aesthetics of the tooth and to provide a tight marginal seal against oral microorganisms. A laboratory‐fabricated crown is often advocated as the optimal restorative treatment for the root filled tooth. Five to ten years after root filling, the survival rates for teeth restored with laboratory‐fabricated crowns are reported to be significantly better than for those restored with composite or amalgam (Ng *et al*. 2010, 2011b, Fransson *et al*. 2016). Although the reported outcomes are in favour of laboratory‐fabricated crowns this may not necessarily imply that they are better than direct restorations in every case. Since the previous studies (Ng *et al*. 2010, 2011b, Spielman *et al*. 2012, Fransson *et al*. 2016, Dawson *et al*. 2017) are not randomized controlled studies selection bias cannot be excluded; teeth with uncertain prognosis may be more likely to receive a direct restoration.

Clinical decision‐making, in general, is a complex process whereby the clinician evaluates clinical findings, patient's requests and medical history (White *et al*. 1992). The decisions are based not only on the clinician's theoretical knowledge and clinical experience of diagnosis and treatment options, but also on other factors such as patient‐related aspects (Wulff & Gøtzsche 2000). Preoperative diagnosis and quality of the root filling may influence the decision on the type of coronal restoration (Chugal *et al*. 2007, Dahlström *et al*. 2018), but little is known about other factors such as patient‐related aspects of the dentist's decision‐making process with respect to the choice of coronal restoration of a root filled tooth.

Research methods for qualitative data are considered useful for analysing people's thoughts, feelings, attitudes, perceptions and preferences and are applied when depth, insight and understanding are required of a particular phenomenon, in its own context (Gill *et al*. 2008, Stewart *et al*. 2008). Thus, a research method for qualitative data can be used to improve our knowledge and understanding of a variety of issues of importance to dentistry such as factors influencing dentists' treatment philosophies or patients' attitudes to regular dental attendance (Gill *et al*. 2008). The aim was to characterize the dentist’s perspective of the decision‐making process underlying the choice of coronal restoration of a root filled tooth.

## Material and methods

### Qualitative content analysis

The present study was based on interviews with GDPs in Sweden at a location chosen by the informant: at the GDP's clinic (*n* = 14) or at the interviewer's office (*n* = 1). The data were analysed using Qualitative Content Analysis (Graneheim & Lundman 2004).

### Research team and reflexivity

The three authors are specialists in Endodontics, working at a Faculty of Odontology. EW is experienced in qualitative research, whereas HF and VD have only limited experience. The preconception was that the improved survival rates reported for root filled teeth restored with crowns may be attributable in part to selection bias. Apart from technical and biological factors, the dentist's decision‐making process underlying the choice of coronal restoration may also be influenced by financial issues and dentist and patient preferences.

### The Swedish context of RCTs and coronal restoration

In Sweden, RCTs and coronal restorations are undertaken primarily by GDPs, working within the public dental health service (PDHS) or the private sector.

All citizens of Sweden ≥24 years old are entitled to a dental care subsidy from the tax‐funded Swedish Social Insurance Agency (SSIA) including a step‐wise high‐cost protection, whereby the patient in the end pays only 15% of dental fees above 15 000 SEK (approximately 1400 Euro). Most dental treatments are covered by the SSIA, provided that certain criteria are met. For example, for a crown restoration to be reimbursable, the defect of the tooth has to be extensive, that is 4 out of 5 surfaces for a premolar/molar and 3 out of 4 surfaces for an incisor/canine (Tandvårds‐ och läkemedelsförmånsverket 2008). Apart from this fee‐for‐service system, patients have the option of subscribing to basic dental care coverage for a fixed fee, through a tax‐funded insurance called Dental Care for Health offered by the PDHS (Andas & Hakeberg 2014). This includes root fillings, composite restoration and single crowns. The dental clinic receives a fixed compensation for each patient. Some patient's needs, such as dental care due to poor health, are covered by other regulations whereby fees for dental care are reduced but with extensive restrictions. For example, fixed prosthodontics is usually not included at a reduced fee. Dental care is free of charge for patients <24 years old.

### Informants

A strategic selection process was conducted of 15 GDPs practicing in Sweden. Two absolute inclusion criteria applied:

- Recent experience of decision‐making about coronal restorations for root filled teeth; thus a self reported view that a detailed narration about the decision‐making process was possible.
- Fluency in Swedish, essential for in‐depth narration during the interview.

**Figure 1:**
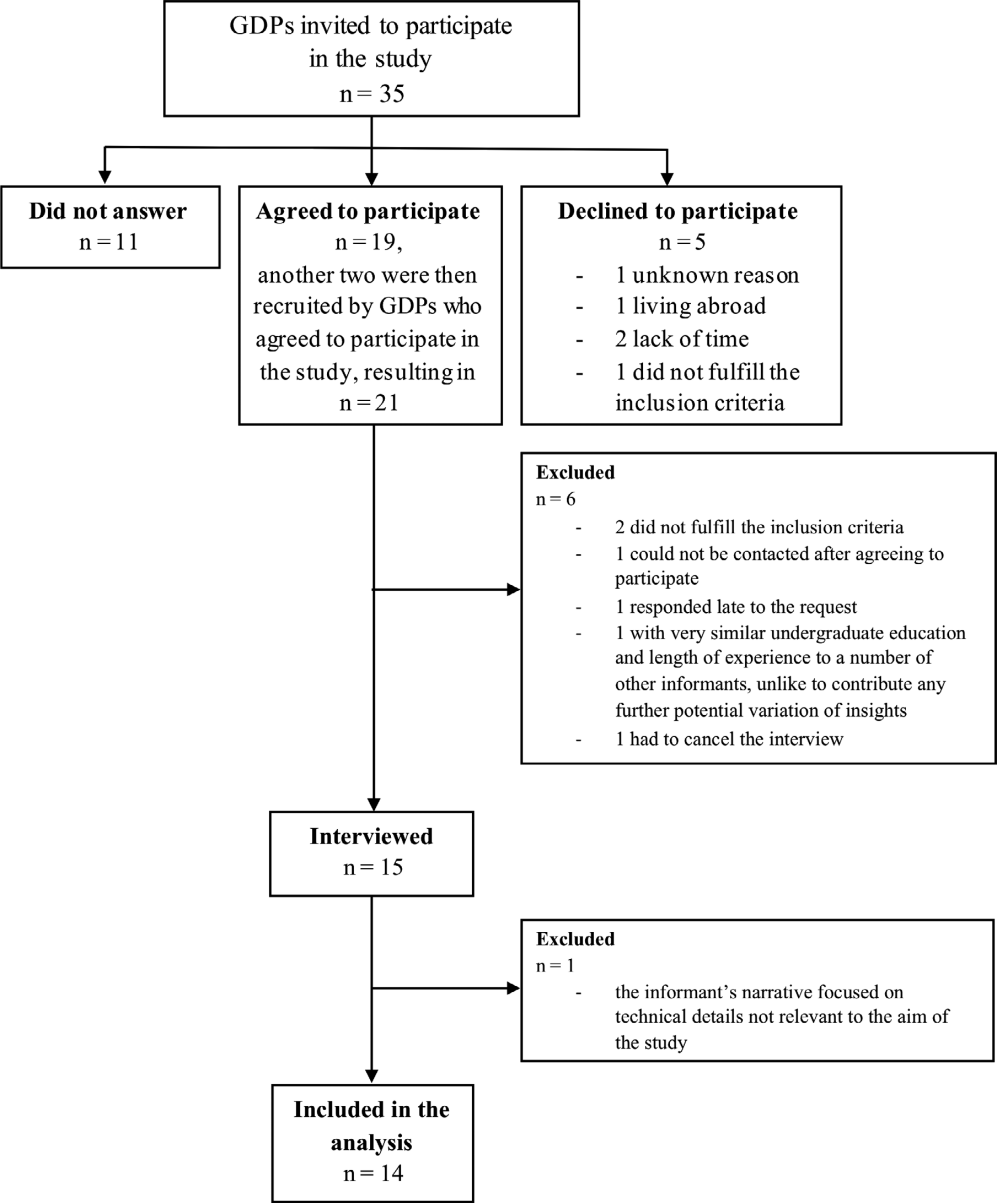
Flow chart of participant recruitment process.

In order to ensure a variety of participating GDPs and thereby different perspectives on the topic, the informants were strategically selected according to the variables in Table 1 also showing the distribution of the selected GDPs. Figure 1 illustrates the process whereby 14 participants were finally included in the study and analysis. Full details about the recruitment, pilot study, data collection, text preparation and data analysis are presented in the Appendix S1. An example of the text preparation process and analysis is presented in the Appendix S2.

**Table 1:** The distribution of 14 informants with respect to gender, age (<35, 35–50 or >50 years), location of undergraduate dental education, service affiliation and length of professional experience

Gender (*n*)	
Male	(8)
Female	(6)
Age, years (*n*)	
<35	(2)
35–50	(7)
>50	(5)
Undergraduate dental education (*n*)	
Gothenburg	(3)
Malmö	(3)
Stockholm	(3)
Umeå	(5)
Service affiliation (*n*)	
Private practice (PP)	(5)
Public Dental Health Service (PDHS)	(9)
Professional experience, years (*n*)	
<10	(5)
10–20	(2)
>20	(7)

The study was approved by the Regional Ethical Review Board at Lund University, Lund, Sweden (Dnr 2016/547). Before the interview the GDPs received verbal and written information about the study and they provided written consent.

## Results

**Figure 2:**
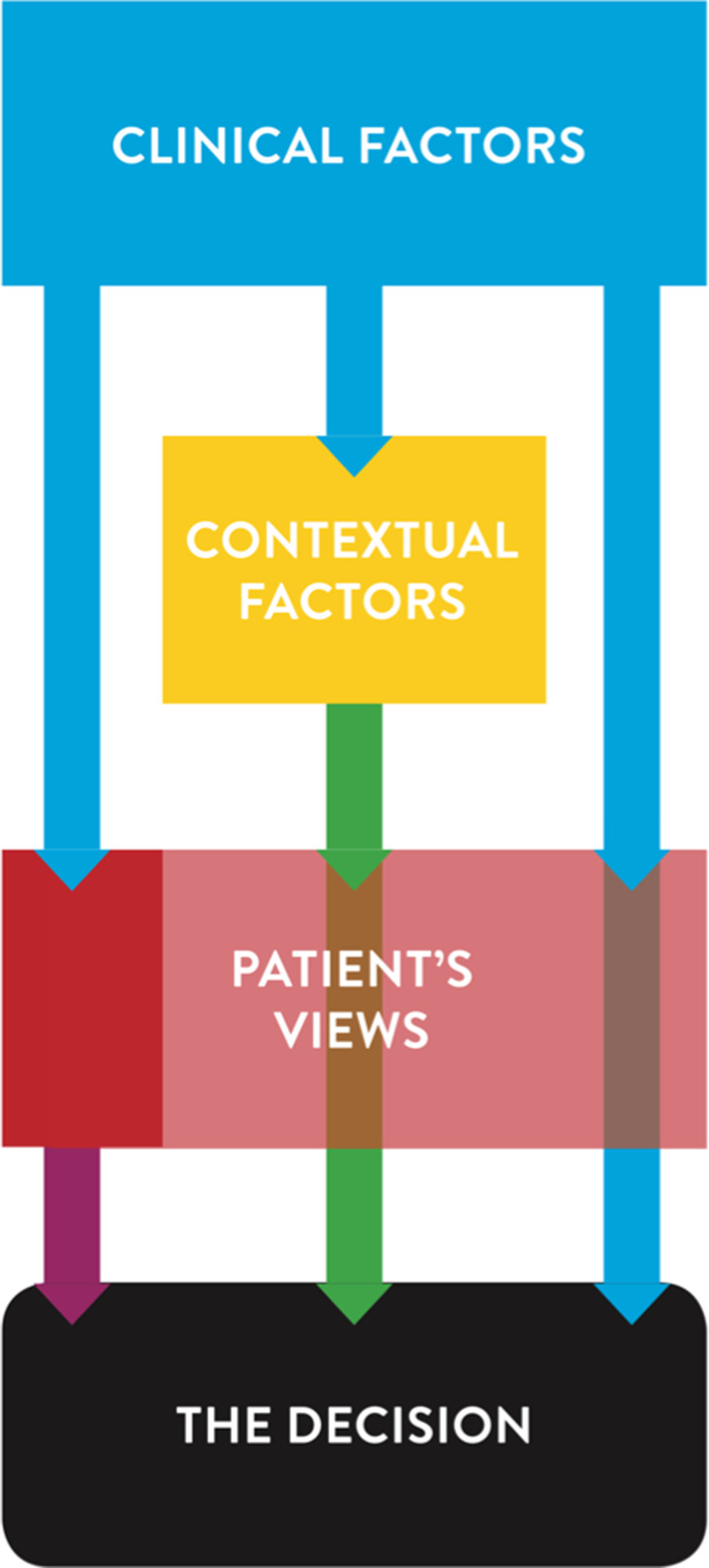
The dentist´s decision process was based on clinical factors (blue arrows). In certain cases, contextual factors were also taken into account and occasionally modified the choice (green arrow), but the patient´s preference was decisive, resulting in either approval (arrows unchanged) or modification (purple arrow) of the dentist´s decision.

One theme covering the latent content was identified: *Clinical factors considered important but overruled by context and patient opinions, if in conflict*. The dentist's decision‐making process was in each case characterized by an assessment of clinical factors for which, in certain cases, contextual factors were taken into account and occasionally modified the choice. However, the patient's views prevailed (Fig. 2). Three main categories were identified, including seven subcategories which constituted the manifest content (Table 2).

**Table 2:** The theme, categories and subcategories with corresponding codes

Theme	Clinical factors considered important but overruled by context and patient opinions, if in conflict		
Category	Clinical factors	Contextual factors	Patient´s view
Subcategory and codes	**Dental status** Major loss of tooth substance favours a crown Poor prognosis favours C A crack is a determinant for choice of crown therapy	**Patient‐related** Dental fear obviously supports choice of C Relationship with patient influences decision	**Mutual decision** Dentist’s recommendation determines patient’s decision Different motives but agreement on appropriate treatment (dentist and patient – C)
	**Longevity assessment** Longevity assessment basis for choice of crown Longevity for C considered good – contributes to decision	**Dentist‐related** Dentist’s preference influences recommendation (always a crown) Dentist’s current state of mind influences decision (chose a crown but could have chosen C instead)	**Compromise** Patient’s wishes determine decision – dentist compromises (C instead of crown) Respect patient´s decision despite different treatment goals Clear information a prerequisite for compromise
		**External circumstances** Lack of time – dentist chooses C Social subsidies support choice of C Social Services regulations determine recommendation for C	

C, composite restoration.

### Clinical factors

Clinical factors, dental status in general and potential longevity of the restoration formed the basis of the GDPs' decision about the type of coronal restoration. Occasionally, one clinical factor was the sole determinant, whilst in certain cases, several factors in combination contributed to the decision. The subcategories dental status and assessment of longevity are described further in Appendix S3.

### Contextual factors

When taken into account, contextual factors could support the GDPs' choice of coronal restoration and thus influence the choice of treatment. Contextual factors could also modify the proposal for restoration: although a crown might be preferable, the GDP could still amend the treatment plan to a composite filling.

#### Patient‐related factors

Factors related to the patient were taken into account. The patient’s age, although not determinant, could support a decision, based on clinical factors, to restore the tooth with a crown:

‘He isn’t so… … No, he was born in −73, or something … I mean he is going to have the use of that tooth for a long time. … And that sort of has some influence … on my reasoning about…, about which therapy we choose’.

The impact of the patients' motivation and interest in their teeth was exemplified by a situation in which a crown was the restoration of choice, because of the extensive loss of tooth structure:

‘[….] so there will be a crown on that tooth. [….] It is a patient who wants to keep her teeth. She is keen for me to do a crown, she is the sort of patient who wants me to do what is best’.

Needs and desires expressed by the patient were also taken into account. One patient requested improved aesthetics and a more durable restoration because the previous composite restorations had failed repeatedly. Based on clinical factors, the GDP considered a crown to be preferable to a composite which was a decision that reflected the patient's need.

Occasionally, the GDP's understanding of a patient's complex situation would influence recommendation of a composite instead of a crown:

‘Because at first she wasn’t very interested in keeping her appointments. [….] In fact she has so many teeth which are doubtful, so that … there’s not enough money to cover that sort of prosthetics. Erm… … … It will be very difficult to make a start, because it’s a bit difficult: Wherever should one start? [….] Getting oral hygiene established…,… erm…, get…, get control of … yes, like active caries lesions …,… and… * Little laugh * … get…, finally finish the root fillings. Because this has taken … We have been working on this for several years. * Laugh *’

Knowledge about the patient's financial status could influence the choice of composite, although a crown in fact would have been better. Dental fear was sometimes also decisive for choosing composite:

‘Is *extremely* anxious during dental treatment [….] She…, … she says this: [….] Are you ready soon? … The rubber dam makes me panic. [….] Lies on the side of the chair. Ehh… Brings a friend along, holds her hand. So … this is difficult. [….] And I knew she wanted to get out of here as soon as possible. [….] Despite [….] … nice surrounding teeth and intact dentition so…, … so I chose a filling for this situation. [….] Despite it actually … … I might have done some type of porcelain restoration which held it together’.

One informant mentioned that the dentist–patient relationship could influence the GDP's decision:

‘ a patient … … who … … says that her previous dentist caused her problem …, … then I always feel a little worried, because then it feels like if *I* were to treat her and she had discomfort afterwards then she would blame me for causing her … problem. Even if perhaps it isn’t what I have done *actually* which …, which is the cause of it. Yes. That it…, that I become a little more … restrictive…, … about… * Little laugh * … about…, what I do. So that… So that is in fact also a …, … … a reason that … from *my* perspective, not to do a crown’.

The informant also commented that this was a new patient and at the time of the decision to do a composite restoration they did not know each other very well. It would be good if they would consider a crown restoration later on.

#### Dentist‐related factors

A desire to preserve sound tooth structure was expressed: to remove and sacrifice healthy tooth structure in crown preparation was regarded as overtreatment:

‘There is quite a small harmless amalgam filling. And there are no cracks or such. And so I think it feels a bit clumsy to … start preparing that tooth afterwards. [….] Because I feel that is a bit like overtreatment …, … when the tooth has such a small filling’.

A somewhat contradictory example was that of a tooth entirely restored with composite, except for the buccal surface. The restorations were considered small and of high quality. The dentist therefore perceived the tooth to be adequate and thought it was wrong to remove sound tooth structure to make a crown.

The informants considered that root filled teeth restored with crowns survived longer, because the crown protects the tooth by encircling it and holding the remaining tooth structure together better than composite.

‘I usually always suggest a crown as the treatment of choice. [….] Yes, it lasts better in the long run, in fact’.

Preference for a crown was also expressed in a general context, the GDP reflecting that for the patient, RCT is uncomfortable, time‐consuming and expensive. As much efforts are put in to a RCT, protection by a crown is justified to increase the survival time. The GDP also commented:

‘And then I also think it's rather fun to do crowns’.

The dentist's current state of mind also had an impact on the decision, in combination with clinical factors:

‘Otherwise, I might well have just done a new filling as well. It wasn't that bad at all. But … my frame of mind that day was… * Laugh * Then the choice was for…, for the crown’.

#### External circumstances

The particular payment model and accompanying rules were mentioned by the informants in conjunction with the decision. They stated that the SSIA's regulations, although not decisive, were often kept in mind as some kind of guideline for the choice of restoration, as clinical factors constituted the basis for the decision. When the GDP decided to recommend a crown it was mentioned that it was in accordance with SSIA's criteria:

‘Yes, it was in fact so that it came under these …, according to the national guidelines. That a certain amount of tooth structure is missing. I believe that for a front tooth there have to be three surfaces missing …, … to be able to do a crown. So it was in accordance with the Swedish Social Insurance Agency …, Yes it did. Mm’.

When composite was chosen, the informants often mentioned that SSIA's criteria would not indicate a crown. However, if the patient qualified for high‐cost protection, some GDPs stated that it facilitated their decision to recommend a crown in cases of uncertain prognosis, because the treatment would not be very expensive for the patient. In the same way as for SSIA's regulations, the rules within the payment model Dental Care for Health served as a guideline for the choice of restoration as well as financial interest for the practice:

‘There has been a lot of fiddling about with that tooth. She has come to me for a number of appointments. [….] And moreover she is covered by Dental Care for Health …[….] I expect that purely financially it would be better for the clinic to do a crown straight away …’.

However, the GDPs' decision about providing a coronal restoration was directly affected by the regulations applying to patients with an increased need for dental care due to poor health. In these circumstances, due to the restrictions for fixed prosthodontics the choice of composite was often obvious, although in the opinion of the GDP a crown would have been a better option. Another external circumstance influencing a GDP's decision was the lack of time available to provide a crown, which was the preferred treatment of choice.

### ***Patient***'***s view***

The GDP's recommendation and the fee for the suggested treatment alternatives were presented to the patient and the reasons for choosing a certain restoration and prognostic information were given. Depending on the patient's response, either consent was obtained, or the decision was modified according to the patient's financial circumstances and preferences, provided that the dentist found it acceptable. If not, an extended discussion was initiated by the dentist, to further explain why a certain treatment was considered necessary.

#### Mutual decision

The dentists' recommendation, based on their knowledge and clinical experience, was often decisive for the patient's decision. When a coronal restoration was suggested by the dentist, there was often not much discussion as the patients readily accepted and agreed, usually without any objections. Most patients were described as being amenable to the treatment recommendation. Trust and a good dentist–patient relationship were stated by some informants to be contributing factors for reaching a mutual decision:

‘He has been a patient here for a long time in fact. He trusts me that I can … what I do, like, and …,…still a good relationship, I think. So that those who have been my patients for a while, well they…, they accept my suggestions, in fact. It…, it is very good’.

If the dentist and patient were unable to agree about the treatment plan, and the dentist found the patient's wishes unacceptable, the dentist initiated an extensive discussion, including a thorough explanation. Situations were mentioned in which patients requested extraction, but in the dentist's view the tooth was not in a condition that warranted extraction, but instead endodontic treatment and subsequent restoration. After such a discussion, patients often changed their views, accepting the dentist's advice and a mutual decision was reached. This extended discussion included whether to retain the tooth or not; once this was agreed upon, few problems were encountered about the decision to have a crown. One informant stated that if the patient had not consented to a crown restoration instead of composite, the informant would have continued with further discussions to persuade the patient otherwise.

Some informants stated that patients under the payment model Dental Care for Health readily agreed to the suggested treatment, and no financial consideration was involved:

‘So she was quite in agreement with having a crown. And moreover she is covered by Dental Care for Health …, … so then there is not much discussion …, … erm…, about the cost of treatment. So I was able to give my opinion. And then she agreed to it’.

Occasionally, several treatment options were possible. Apart from a crown restoration as part of a fixed partial denture to replace a missing tooth, implants and removable partial dentures were alternatives. For some teeth, both composite and crowns were considered suitable. The options were presented to the patient, who then made the choice, so that a mutual decision was reached.

#### Compromise

When the patient was unwilling to accept the dentist's suggestion for a coronal restoration, the dentist took the patient's view into account and modified the decision accordingly. This was the case when a crown was considered to be the best treatment option, but composite was chosen instead. Modifying factors would include the patient’s finances as well as personal preferences. For example, as expressed in the quote below, one patient just wanted to keep her tooth, infection‐free, without having it restored to function.

‘There is also major loss of tooth substance. Here I would be very, very happy to put a crown on this tooth. But she thinks it feels unnecessary and expensive. [….] But rather she wants me to rootfill the tooth and just … … like put a small cover on the top. Mm. And you also have to … accept that’.

The informants expressed understanding and respect for the patient's preference for a composite instead of a crown restoration, irrespective of their own opinion about the choice:

‘Of course I can understand this, when one is so old and …, 80 years of age, and then … still, it is so delicate that there is no great stress …, … then I think that…, really there is no …, ver…, very important reason to prefer a crown. It functions well just the same’.

However, the dentists did not accept a compromise decision without reflection. When they agreed to composite restoration instead of a crown, it was preceded by an assessment of longevity. In this context the dentist considered the compromise to be acceptable:

‘It was much for financial reasons that we did composite crowns. … … [….] in fact she didn’t have a very …, not very strong jaws, instead she looked quite …, … not such a heavy *bite*. And so I felt quite confident to be doing plastic crowns. It might not last a lifetime, but … yes, just the same it will function well for quite a long time’.

In conjunction with the compromise decision, the patient was informed of the prognosis, and that a filling was assumed to survive in the short term and not the long term. Furthermore, the risk of fracture would also be greater.

Another circumstance in which a compromise decision was made occurred when the tooth in question was in fact in such poor condition that the GDP recommended extraction. In patients unwilling to consent to extraction, the dentist compromised and agreed instead to provide RCT. In such cases, composite was the obvious choice for restoration.

## Discussion

The GDP's decision‐making process in restoring a root filled tooth is complex and based on diverse factors. Clinical factors were considered important but could be overruled by context and patient opinions, if the patient and dentist disagreed. In each case, the dentist's decision‐making process was characterized by an assessment of clinical factors for which, in certain cases, contextual factors were taken into account, and occasionally modified the choice. However, the patient's views were decisive.

Selection of informants was strategic in order to achieve different perspectives on the dentists’ decision‐making process about coronal restoration, using the maximum variation sampling strategy (Swedish Agency for Health Technology Assessment & Assessment of Social Services (SBU) 2014). The selected informants were diverse and the results may be considered applicable to Swedish GDPs.

By the use of semistructured interviews comprising mainly open ended questions, the informants were invited to express themselves freely on the topic, using their own authentic cases as a basis for the narration. This was considered to strengthen credibility, as it was possible to study the GDPs decision process in context. Furthermore, the GDPs were allowed to narrate from their perspective, disclosing which factors had influenced their choice of coronal restoration, whilst the interviewer's presence was kept at a minimum, increasing the conformability.

Some informants might have perceived the interviewer, a specialist in endodontics, as an ‘authority’, and potentially critical of the GDP's decisions. Thus, some informants may have modified their narrative to please the interviewer. However, before the interview the GDPs were informed about the lack of knowledge on this topic and that the informant's specific experience would provide valuable information.

The number of informants required in a study using a qualitative approach depends on the quality of the data obtained and the complexity of the phenomena being studied (Graneheim & Lundman 2004). For the present study, data from 14 informants were considered sufficient to address the research question, as saturation had been achieved.

The GDP’s decision‐making process involved a number of factors. After clinical assessment, an evaluation was made, followed by a discussion with the patient and then a decision. In some cases the GDP’s decision was simple. In other cases the decision was more complex, involving not only clinical factors but also different contextual factors. The clinical factors considered in decisions about coronal restoration are in accordance with those described in textbooks and clinical guidelines, which involve considering the amount and quality of remaining tooth structure, the position of the tooth in the arch, anatomy and function (Gulabivala & Ng 2018, Zarow *et al*. 2018).

The technical quality of the root filling was not mentioned as having any great impact on choice of restoration, a somewhat unexpected finding as it is a factor of importance in the outcome of the RCT (Ng *et al*. 2008). The reason why root filling quality was not mentioned more often is unknown. In a recent study, it was found that GDPs view RCTs as complex and difficult but also ‘illogical’, as successful outcomes are sometimes achieved despite the poor quality whilst some failures occur in cases of adequate quality (Dahlström *et al*. 2017). Even though root filling quality was rarely mentioned some informants commented that a crown seemed to be a more appropriate choice in the case of an adequate root filling. Another interesting issue concerned teeth root filled by colleagues. For all informants, the decision about coronal restoration was preceded by an assessment of the technical quality of the root filling, followed by a decision to retreat when it was deemed inadequate. It is possible that the GDPs found it more comfortable, in discussion with a specialist in endodontics, to comment on the quality of root fillings done by their colleagues rather than their own. However, the results imply that the prognosis of the RCT influences the decision, but to what extent is unknown.

The importance of the context in which the decision about coronal restoration was made, was a significant finding. Although clinical factors were important, they were not always decisive. Patients suffering from dental phobia, the dentist's current frame of mind, lack of time and payment model are examples of contextual factors that overruled clinical factors, leading the GDP to recommend composite instead of a crown. In the same way, the patient's preferences could also overrule clinical factors, sometimes resulting in a compromise decision. This is in accordance with a previous study, reporting that not only disease status was important, but that dentists' personal views, patients' preferences, professional fees/time and ethical aspects also had an impact on restorative treatment decisions (Kay & Blinkhorn 1996).

The results of the present study support the preconception, that selection bias may account, at least in part, for the favourable outcomes reported for root filled teeth with indirect restorations. In the present study some teeth were obviously less likely to be restored with a crown, despite meeting clinical indications: uncertain prognosis, patients with low motivation or poor economic status, or a particular payment model which did not cover crowns. Thus, the higher extraction rates of teeth with composite (Ng *et al*. 2010, 2011b, Landys Borén *et al*. 2015, Fransson *et al*. 2016) may be attributable to factors other than the type of restoration.

Systematic reviews of the literature have highlighted the lack of scientific evidence which determines whether indirect restorations maintain the longevity of a root filled tooth better than a direct restoration (Swedish Agency for Health Technology Assessment & Assessment of Social Services (SBU) 2010, Sequeira‐Byron *et al*. 2015). However, a crown restoration is often advocated as the optimal restorative treatment for the root filled tooth. This view was expressed by some GDPs in the present study, either explicitly or implicitly, which is in accordance with a previous study (Rotstein & Salehrabi 2008). Decisions in favour of a crown were always motivated in terms of clinical factors, but a dentist's preference for a crown may have facilitated the choice. On the whole, Swedish GDPs restore more root canal treated teeth by direct (57.7%) than indirect (22.5%) restoration (Dawson *et al*. 2017).

Insurance and reimbursement regulations were not described as a direct reason for choosing a certain restoration but had an indirect impact on the decision. None of the GDPs mentioned cases where a crown was chosen, even though the SSIA's criteria had not been fulfilled. As the crown will not be reimbursed by the SSIA the GDPs may be more likely to suggest composite in such cases. However, although the criteria for a crown were met, it was not always the chosen treatment.

The GDPs' decision about coronal restoration always took into account the patient's views, which had a significant impact. The GDPs stated that patients usually agreed to the suggested treatment. Trust and confidence in the dentist were expressed as factors facilitating the patient's decision. This has been reported in earlier studies as the most important factors influencing acceptance of the proposed treatment plan by patients (Oates *et al*. 1995). Other factors influencing the patients' decision include fees, past dental history and pain and discomfort associated with treatment (Kalsi & Hemmings 2013). When patients did not accept the recommendation of a crown the informants usually associated this with economic factors. The association between patients' financial status and choice of coronal restoration after RCT has been demonstrated previously (Olsson *et al*. 2019).

## Conclusion

In Sweden, GDPs reported that their decision‐making process, leading to the choice of coronal restoration of a root filled tooth, was based not only on clinical factors but also on decisive contextual factors and the patients’ views. In each case the decision‐making process was characterized by an assessment of clinical factors for which, in certain cases, contextual factors were taken into account, occasionally modifying the choice. However, the patient's views were ultimately the decisive factor.

## Conflicts of interest

The authors have stated explicitly that there are no conflicts of interest in connection with this article.

## Supporting information


**Appendix S1.** Materials and methods: recruitment, pilot study, data collection, text preparation process and data analysis.

**Appendix S2.** The text preparation process. An example of a meaning unit with its corresponding condensed form, code, subcategory and category.

**Appendix S3.** Results: the subcategories dental status and assessment of longevity.
