# Supplementary material for: Coronal restoration of the root filled tooth – a qualitative analysis of the dentists' decision‐making process
Source: Int Endod J. 2020 Dec 4;54(4):490–500. doi: 10.1111/iej.13442 (PMC7983980; doi:10.1111/iej.13442)
Supplement: Supplementary file 1 — Appendix S1. Materials and methods: recruitment, pilot study, data collection, text preparation process and data analysis. [file IEJ-54-490-s001.docx]

**Appendix S1. Material and methods: recruitment, pilot study, data collection, text preparation process and data analysis.**

*Recruitment*

Through personal contacts, informants were successively recruited and interviewed, until saturation was considered to be achieved. In total, 35 GDPs were contacted, either by telephone or e-mail (Fig. 1). They were given information about the study, the interviewer´s professional status as a specialist in Endodontics and a PhD-student and invited to participate. Before the interview, the GDPs who agreed to participate were contacted by the interviewer by telephone and an interview, face to face, was scheduled. They were asked to prepare an account of their four most recent cases of permanent restoration of a root-filled tooth: two restored with composite and two with laboratory-fabricated crown. Furthermore, these cases were to involve teeth which had been root-filled by the GDPs themselves and had not been previously restored with a laboratory-fabricated crown. If the GDP considered it helpful, anonymized records, radiographs and plaster study models could be brought to the interview.

*Pilot study and test interviews*

Before study start, a pilot study, involving three interviews, was conducted. Two test interviews with feed-back on the interview technique and transcription were conducted by VD before the start of the study. These five interviews were not included in the analysis of the present study.

*Data collection*

In-depth, semi-structured interviews were conducted, focusing on the informant´s personal experience of the process which leads to a decision as to how to restore a root-filled tooth. The interviewer invited the informants to express themselves freely on the topic. During the interview the informants were asked to describe the latest two occasions on which:

- A laboratory-fabricated crown was chosen for permanent restoration of a tooth after RCT.
- A direct composite restoration was chosen for permanent restoration of a tooth after RCT.

The topics covered during the interview remained unaltered during the collection period. The interviewer asked open-ended questions intended to encourage the informant to develop, specify, deepen and reflect on the narrative: *can you tell me more about….?, you said a large amount of residual tooth structure, what does large mean to you?* Questions were also redirected to cover the decision-making process in the specific patient cases, when informants tended to talk more in general about coronal restoration of root-filled teeth. Before the interview was concluded the informants were invited to add any further comments.

The interviews were conducted between October 2017 and October 2018 by one interviewer (VD). Each informant was interviewed on one occasion. The duration was 15 – 40 minutes and the interviews were digitally recorded. The interviews continued until no new information was forthcoming from the informants. One interview was excluded from the text preparation process and analysis, because the informant’s narrative focused on technical details which were not relevant to the study.

*Text preparation process*

Fourteen interviews were transcribed verbatim by a contracted secretary while one author (VD) checked and complemented for non-verbal expressions. The informants did not comment on the transcripts. The transcripts were read several times to gain an overall impression of the material. The interviews were then separated into meaning units; when there was a change in the meaning of what the informant was describing, a mark was made in the text. Then the text of each unit was condensed into a shorter formulation by removing the excess words, while preserving the meaning. For the first interview, identification of meaning units and the condensation were undertaken by two of the authors (VD, EW) together. Thereafter one author (VD) undertook this process for the remaining interviews.

*Data analysis*

Meaning units related to the decision process underlying the choice of coronal restoration were identified and selected for analysis. Through abstraction of these condensed meaning units, they were given a code, reflecting the possible inherent meaning of the content. The first part of one interview was coded together by two of the authors (VD, EW). The remainder of the first interview and a further two more interviews were then coded separately by all authors and compared and discussed until consensus was reached. For the remaining interviews the coding was conducted by the first author (VD). In cases of uncertainty about the codes a discussion was held until consensus was reached.

When the coding process was completed, the emergent patterns, (codes sharing a commonality), were grouped into categories and sub-categories illustrating the manifest level of content (the visible, obvious components). This was followed by identification of a theme illustrating the latent (interpretation of the underlying meaning) level of content. With respect to codes, sub-categories, categories and theme, consensus among the authors was achieved by discussion. The informants did not provide feedback on the findings. An example of the text preparation process and analysis is presented in Appendix S2.
