# Supplementary material for: Coronal restoration of the root filled tooth – a qualitative analysis of the dentists' decision‐making process
Source: Int Endod J. 2020 Dec 4;54(4):490–500. doi: 10.1111/iej.13442 (PMC7983980; doi:10.1111/iej.13442)
Supplement: Supplementary file 2 — Appendix S2. The text preparation process. An example of a meaning unit with its corresponding condensed form, code, subcategory and category. [file IEJ-54-490-s002.docx]

**Appendix S2. The text preparation process. An example of a meaning unit with its corresponding condensed form, code, sub-category and category.**

| **Meaning unit** | **Condensed meaning unit** | **Code** | **Sub-category** | **Category** |
| --- | --- | --- | --- | --- |
| Er… Yes, but it was in fact … fr… One was fractured at the gingival margin. And so it was prosthetic therapy. There was like nothing to …,… to build on. * Little laugh * There was, to be sure, no … tooth substance remaining.… And the other one was also … It had an apical periodontitis and major loss of tooth substance. On both of them. | Fractured at the gingival margin, so it was prosthetic therapy. Nothing to build on. No remaining tooth substance. The other one also had apical periodontitis, and major loss of tooth substance. | Obvious decision for a crown on the basis of extensive loss of tooth substance | Dental status | Clinical factors |
