# Supplementary material for: Coronal restoration of the root filled tooth – a qualitative analysis of the dentists' decision‐making process
Source: Int Endod J. 2020 Dec 4;54(4):490–500. doi: 10.1111/iej.13442 (PMC7983980; doi:10.1111/iej.13442)
Supplement: Supplementary file 3 — Appendix S3. Results: the subcategories dental status and assessment of longevity. [file IEJ-54-490-s003.docx]

**Appendix S3. Results:** the subcategories dental status and assessment of longevity.

Several clinical factors were considered in the decision about coronal restoration, relating to the status of the root-filled tooth as well as neighboring teeth such as the amount and quality of remaining tooth structure, accessibility, tooth group and prognosis. The expected survival of a restoration was also a clinical factor taken into account.

*Dental status*

The status of the tooth to be restored and that of the surrounding dentition, were in each case assessed by the dentist. All informants mentioned the amount of remaining tooth structure as a factor influencing the decision. In cases of a substantial amount of residual tooth structure, according to the GDP´s view, a composite restoration was chosen. Such teeth were described as being intact or having at the most two to three previously restored surfaces, apart from the endodontic access cavity preparation, making the decision to use composite self-evident:

”Because the tooth is actually … unrestored. Except the acce…, access cavity preparation. So in that case, I do not do any prosthetic therapy, rather …, … it is a filling. A simple occlusal filling on it.”

The presence of a crack overruled the amount of remaining tooth structure. Then a crown was preferred, as protection from further damage. When the loss of tooth substance was considered extensive, the GDPs´ decision tended towards a crown.

Few informants mentioned the quality of the endodontic treatment and its potential impact on the choice of coronal restoration. When the quality of the endodontic treatment was mentioned, few cases concerned root-fillings done by the informant but by a colleague. Situations were described in which the GDPs were not completely satisfied with RCT by the informant or by colleagues: in their opinion the technical quality of the root-filling was suboptimal. The unsatisfactory quality contributed, along with clinical and contextual factors, to incline the dentist to recommend a composite restoration. Some informants stated that adequate root-filling quality supported their preference for a crown:

“And then you feel a little more motivated to put a …,… a *crown* on it I think. [….] If you have a good foundation then it …, it feels more … … Er…, it feels in fact easy …, or better to build a …, a good cover over a good foundation.”

Uncertainty about the prognosis of endodontic retreatment was expressed as a decisive reason for choosing composite, although the remaining tooth structure was considered limited:

“In the six (first molar) [….] There was an *enormous* composite on it [….] There was such extensive … periapical destruction. [….] And so we root-filled it like that. Now we have to see whether that large periapical area disappears. And in this case I just did an occlusal composite, because …, I want to see if it heals over time.”

The status of the remaining dentition influenced the decision. A crown was the restoration of choice for a root-filled tooth which was to serve as an abutment in a fixed partial denture. The clinical status of neighboring teeth was also reported to have an impact on the GDPs´ decision. Thus, good status and the need to function as an abutment weighed heavily in favour of a crown while in a heavily restored dentition a composite restoration was preferred.

It was considered difficult to achieve satisfactory aesthetics when only one anterior tooth was to be restored with a crown and this favoured the choice of composite. Other clinical considerations included tooth type (molars) which supported a crown decision, whereas in patients with high caries activity composite was preferred. Perceived technical difficulties could shift the decision towards crowns in some cases and composites in others. Teeth with so little remaining tooth structure that bonding and retention of composite would not be possible, and teeth inaccessible for crown preparation due to their position (distally) were examples of such situations.

*Assessment of longevity*

The choice of composite was preceded by an assessment of expected survival. A stable dentition and the absence of bruxism were mentioned as favourable factors when composite was chosen, as well as the position of the tooth. Anterior teeth are subject to lower forces, and this was considered advantageous for survival of the composite. The longevity of previous restorations was also mentioned:

“So there *can* be a crown … in the future, for this 44. But … no. Not at present. [….] And they still seem to last in her mouth so that, … [….] We have done loads of fillings. * Little laugh *”

When loading forces were considered extensive, it was predicted that a composite restoration would fail and a crown was preferable. This was also the case when a composite filling had already failed.

“And then I also took his bite into consideration, as he lacks both second and third molars…,… that it is…, it should take a lot of load. … … So from a pure durability…point of view so…,… we thought then that the only reasonable thing was … to put a crown on it.”

A somewhat contradictory situation arose in the case of already heavily restored maxillary incisors. As a crown was not considered to be durable without a post and core, composite was chosen to avoid the more advanced treatment.
